# Refeeding syndrome in a 12‐year‐old girl with distal renal tubular acidosis

**DOI:** 10.1002/ccr3.3270

**Published:** 2020-08-20

**Authors:** Yoshiaki Sasaki, Yuichi Akaba, Hiroki Kajino

**Affiliations:** ^1^ Department of Pediatrics Abashiri‐Kosei General Hospital Abashiri Japan

**Keywords:** distal renal tubular acidosis, eating disorder, hypokalemia, refeeding syndrome, serum phosphorus

## Abstract

Distal renal tubular acidosis is a risk factor for refeeding syndrome. Frequent measurement of serum phosphorus levels at the initiation of nutrition and rapid administration of phosphate preparations are required to prevent organ failure.

## Graphical abstract

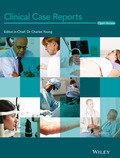

## 1 INTRODUCTION

Refeeding syndrome (RFS) is caused by electrolyte and fluid movement upon resuming nutrition orally, enterally, or parenterally in severely undernourished patients, such as those with eating disorders.^1^ Distal renal tubular acidosis (dRTA) is defined as decreased acid excretion from the distal tubules despite metabolic acidosis and a normal or mildly reduced glomerular filtration rate. We encountered a case of RFS in a patient with an underlying eating disorder and dRTA.

## 2 CASE HISTORY

The patient reported here and her patients have agreed to the publication of this case report.

**FIGURE 1:**
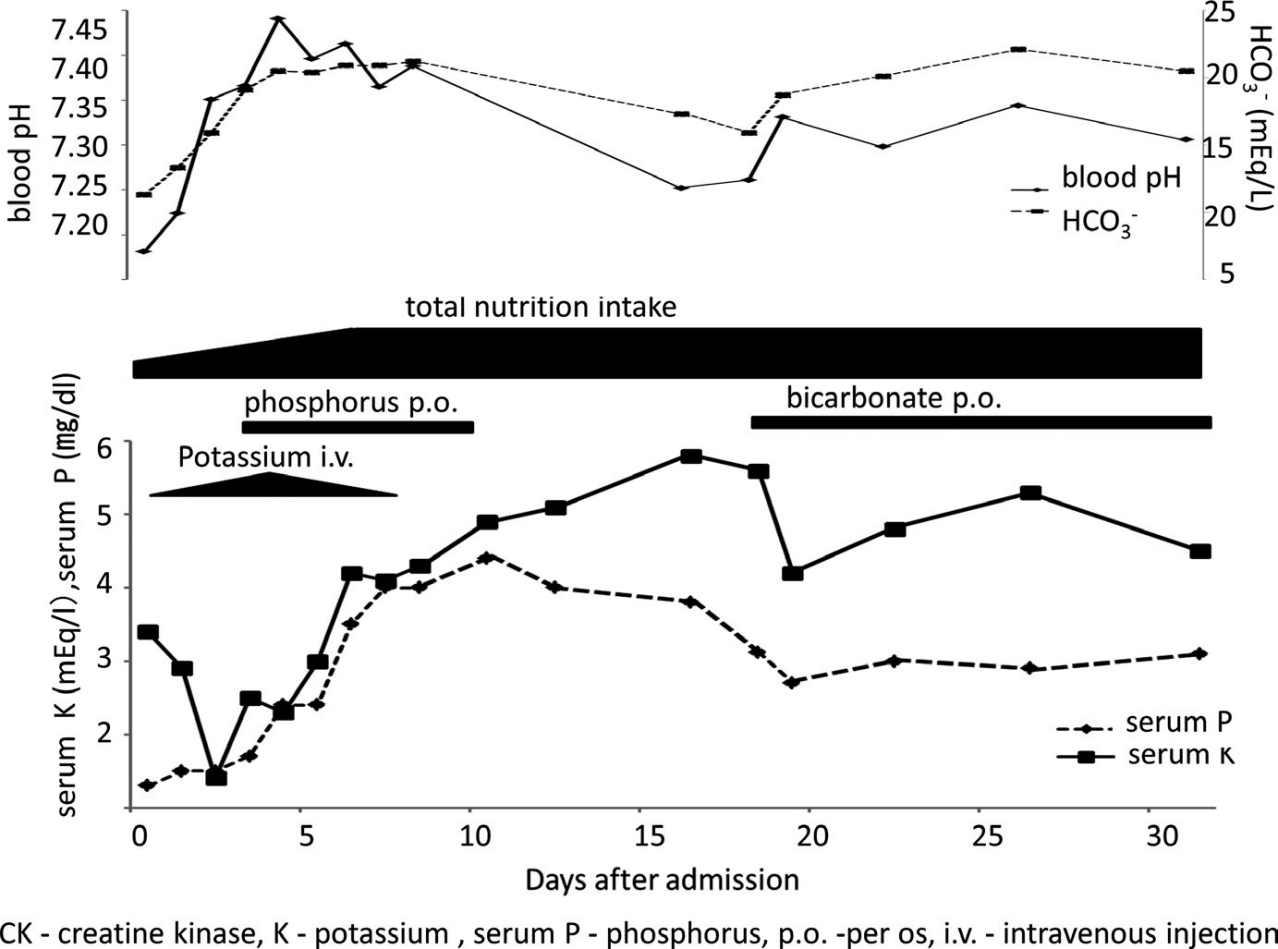
Clinical course of a 12‐y‐old girl serum K—serum potassium, serum P—serum phosphorus, CIV—continuous intravenous infusion

A 12‐year‐old girl visited the emergency department after vomiting for 3 days and generalized muscle weakness. She had neither growth retardation nor previous episodes of muscle weakness, although she had one previous episode of frequent vomiting. However, she had an eating disorder that restricted her diet because of bullying at school 6 months earlier and had observed a 3.4 kg weight loss. As her serum potassium level was 1.3 mEq/L, she was hospitalized with an initial diagnosis of hypokalemic quadriplegia. Other blood test findings were as follows: serum sodium, 148 mEq/L; serum chloride, 126 mEq/L; serum magnesium, 3.6 mg/dL; estimated glomerular filtration rate, 94.9 mL/min/1.73 m^2^; and normal thyroid function (thyroid‐stimulating hormone, 1.193 µIU/mL; free triiodothyronine, 1.72 pg/mL; free thyroxine, 0.94 ng/dL). Her blood pressure was 125/88 mm Hg without diminished cutaneous turgor. Her body mass index (BMI) was 13.5 kg/m^2^ (height, 140.3 cm; weight, 26.7 kg). Blood gas analysis upon admission revealed metabolic acidosis with a pH of 7.181, pCO2 level of 31.3 mm Hg, base excess of −16.3 mmol/L, and normal anion gap (corrected for the serum albumin level) of 10.2 mEq/L. Her vitamin B1 level was 44 ng/mL. After admission, treatment was initiated with potassium infusion of up to 0.2 mEq/kg/h and total calorie intake of 300 kcal/d by parenteral nutrition. The patient's vomiting and generalized muscle weakness resolved, and there was improvement in hypokalemia and metabolic acidosis. However, on Day 3, her serum phosphorus level decreased to 1.4 mg/dL while her creatine kinase (CK) level increased to 7726 U/L (Figure 1). Therefore, we diagnosed RFS with rhabdomyolysis. To treat RFS, oral phosphate administration (900 mg/d of dibasic sodium phosphate anhydrous) was initiated. The serum phosphorus levels normalized on Day 6. Serum CK peaked on Day 4 (12 439 U/L) but normalized on Day 7. Potassium correction continued until Day 7, and oral phosphate administration continued for an additional 3 days. She was discharged after 31 days in hospital with no observed organ failure. In this case, the patient's metabolic acidosis had a normal anion gap, her urinary beta 2‐microglobulin level was high (64 567 µg/L), and her blood pH was lower than 7.30. The patient's urine pH did not fall below 5.5. Her urinary anion gap was 19.9 mEq/L which suggested the presence of distal renal tubular acidosis (dRTA) and rules out laxative abuse. Moreover, we found that her HCO3‐excretion fraction with sodium bicarbonate load was 2.16% (normal: <3%). Her condition was complicated with dRTA. At the last follow‐up, neither nephrocalcinosis nor sensorineural hearing loss had been observed. Oral intake of sodium bicarbonate and potassium citrate preparations has been continued.

## 3 DISCUSSION

In general, when sugar is rapidly supplied to an undernourished patient, insulin is simultaneously released with phosphate, potassium, and magnesium which are taken into the cell, resulting in hypophosphatemia.^1^ As a result, 2,3‐diphosphoglycerate levels in erythrocytes are reduced and the oxygen affinity of hemoglobin is lowered, leading to reduced oxygen supply to peripheral tissues.1 RFS reportedly occurs in 10%‐40% of undernourished patients.^2^ Complications associated with RFS include heart, respiratory and/or renal failures, and evidence of skeletal muscle, nervous system, endocrine, and/or blood disorders.^3^

In this case, hypokalemia developed because of an underlying eating disorder beginning from 6 months prior to admission and latent dRTA. Hypokalemia and low BMI have been previously reported as high‐risk factors for RFS development.^3^ To prevent RFS, healthcare professionals must recognize the possibility of a patient to develop RFS and understand the risk factors from the introduction of nutrition therapy. Patients must be monitored frequently, and in case of low serum phosphorus levels, phosphoric acid preparations must be administered promptly.^4^

At the beginning of treatment, oral intake was difficult for our patient because of nausea; therefore, parenteral nutrition was initiated with a total caloric value of 300 kcal/d (approximately 11.5 kcal/kg/d). This was in line with the recommended dose for patients at high‐risk for RFS at the start of nutrition, which is 5‐15 kcal/kg/d.^5^ The patient's calorie intake was carefully increased, although RFS developed nonetheless. However, no severe organ failure related to RFS was observed because of the early recognition of hypophosphatemia and the prompt initiation of orally administered phosphate preparations.

In conclusion, patients with a low BMI may be more likely to develop RFS when there is an underlying disease, such as dRTA. Therefore, frequent measurement of serum phosphorus levels at the initiation of nutrition and rapid administration of phosphate preparations are required to prevent RFS‐related organ failure.

## CONFLICT OF INTEREST

None declared.

## AUTHOR CONTRIBUTIONS

YS: wrote the manuscript. YA: performed in‐hospital care for this case. HK: critically reviewed the manuscript. All authors: read and approved the final manuscript.

## References

[1] Crook MA . Refeeding syndrome: problems with definition and management. Nutrition. 2014;30:1448‐1455. 25280426 10.1016/j.nut.2014.03.026

[2] Walmsley RS . Refeeding syndrome: screening, incidence, and treatment during parenteral nutrition. J Gastroenterol Hepatol. 2013;4:113‐117. 10.1111/jgh.12345 24251716

[3] National Collaborating Center for Acute Care . Nutrition Support for Adults: Oral Nutrition Support, Enteral Tube Feeding and Parenteral Nutrition. Clinical Guideline CG32, London, UK: National Collaborating Center for Acute Care; 2006. 21309138

[4] Afzal NA , Addai S , Fagbemi A , Murch S , Thomson M , Heuschkel R . Refeeding syndrome with enteral nutrition in children: a case report, literature review and clinical guidelines. Clin Nutr. 2002;21:515‐520. 12468372 10.1054/clnu.2002.0586

[5] Reber E , Friedli N , Vasiloglou MF , Schuetz P , Stanga Z . Management of refeeding syndrome in medical inpatients. J Clin Med. 2019;8:2202. 10.3390/jcm8122202 PMC6947262 31847205

